# SeizureBiomeDB: a unified database of gut microbiome alterations in pediatric and adult epilepsy

**DOI:** 10.1093/bioadv/vbag198

**Published:** 2026-08-06

**Authors:** Zehaan Walji, Parshva Dave, Meherab Ali, Omar Negmeldin, Armaan Kaur Toor, Jane Shearer, Chunlong Mu

**Affiliations:** Department of Computer Science, University of Calgary, Calgary, AB T2N 1N4, Canada; Department of Biological Science, University of Calgary, Calgary, AB T2N 1N4, Canada; Department of Computer Science, University of Calgary, Calgary, AB T2N 1N4, Canada; Department of Biological Science, University of Calgary, Calgary, AB T2N 1N4, Canada; Department of Neuroscience, University of Calgary, Calgary, AB T2N 1N4, Canada; Faculty of Kinesiology and Department of Biochemistry and Molecular Biology, Cumming School of Medicine, University of Calgary, Calgary, AB T2N 1N4, Canada; Department of Neuroscience, University of Calgary, Calgary, AB T2N 1N4, Canada; Faculty of Kinesiology and Department of Biochemistry and Molecular Biology, Cumming School of Medicine, University of Calgary, Calgary, AB T2N 1N4, Canada; Department of Microbiology, Immunology, and Infectious Diseases, Cumming School of Medicine, University of Calgary, Calgary, AB T2N 1N4, Canada; Faculty of Kinesiology and Department of Biochemistry and Molecular Biology, Cumming School of Medicine, University of Calgary, Calgary, AB T2N 1N4, Canada; Hotchkiss Brain Institute, Cumming School of Medicine, University of Calgary, Calgary, AB T2N 4N1, Canada; Alberta Children’s Hospital Research Institute, University of Calgary, Calgary, AB T2N 1N4, Canada; Faculty of Kinesiology and Department of Biochemistry and Molecular Biology, Cumming School of Medicine, University of Calgary, Calgary, AB T2N 1N4, Canada; Department of Microbiology, Immunology, and Infectious Diseases, Cumming School of Medicine, University of Calgary, Calgary, AB T2N 1N4, Canada; Hotchkiss Brain Institute, Cumming School of Medicine, University of Calgary, Calgary, AB T2N 4N1, Canada; Alberta Children’s Hospital Research Institute, University of Calgary, Calgary, AB T2N 1N4, Canada

## Abstract

**Motivation:**

The gut microbiome plays a critical role in health, immunity, metabolism, and brain function. Growing evidence links it to neurological health via the gut–brain axis. Seizure disorders are associated with distinct microbial changes that vary by epilepsy type and developmental stage, yet data are scattered across numerous published studies. To address this, we developed SeizureBiomeDB, a curated, open-access database that compiles research on gut microbiome alterations in epilepsy across animal models and clinical populations.

**Results:**

A comprehensive manual literature search was performed across PubMed, Scopus, Embase, and the Web of Science, incorporating studies with case-control designs, animal experiments, and therapeutic interventions. Extracted data included demographics, seizure type, study design, methodology, and key findings related to the microbiome. The database was built using React and PostgreSQL for secure and efficient data management. SeizureBiomeDB integrates findings from over 80 studies covering pediatric, adult, and animal models. It is accessible via a global, searchable web platform. Interactive visualization tools, including an interactive heatmap and bar graphs, enhance data exploration and cross-study comparisons. To aid interpretation, microbial taxonomy (over 240 taxa) and function are linked to resources such as BacDive and MiMeDB. SeizureBiomeDB offers a structured hub to explore gut microbiome shifts across seizure subtypes and taxa levels. With robust filtering, users can easily extract relevant insights, fostering hypothesis generation for therapeutic targets. This platform underscores the significance of a centralized knowledge database in facilitating current advancements in gut microbiome research and epilepsy. Database URL: https://www.seizurebiome-db.ca/.

## Graphical Abstract

**Graphical Abstract:**
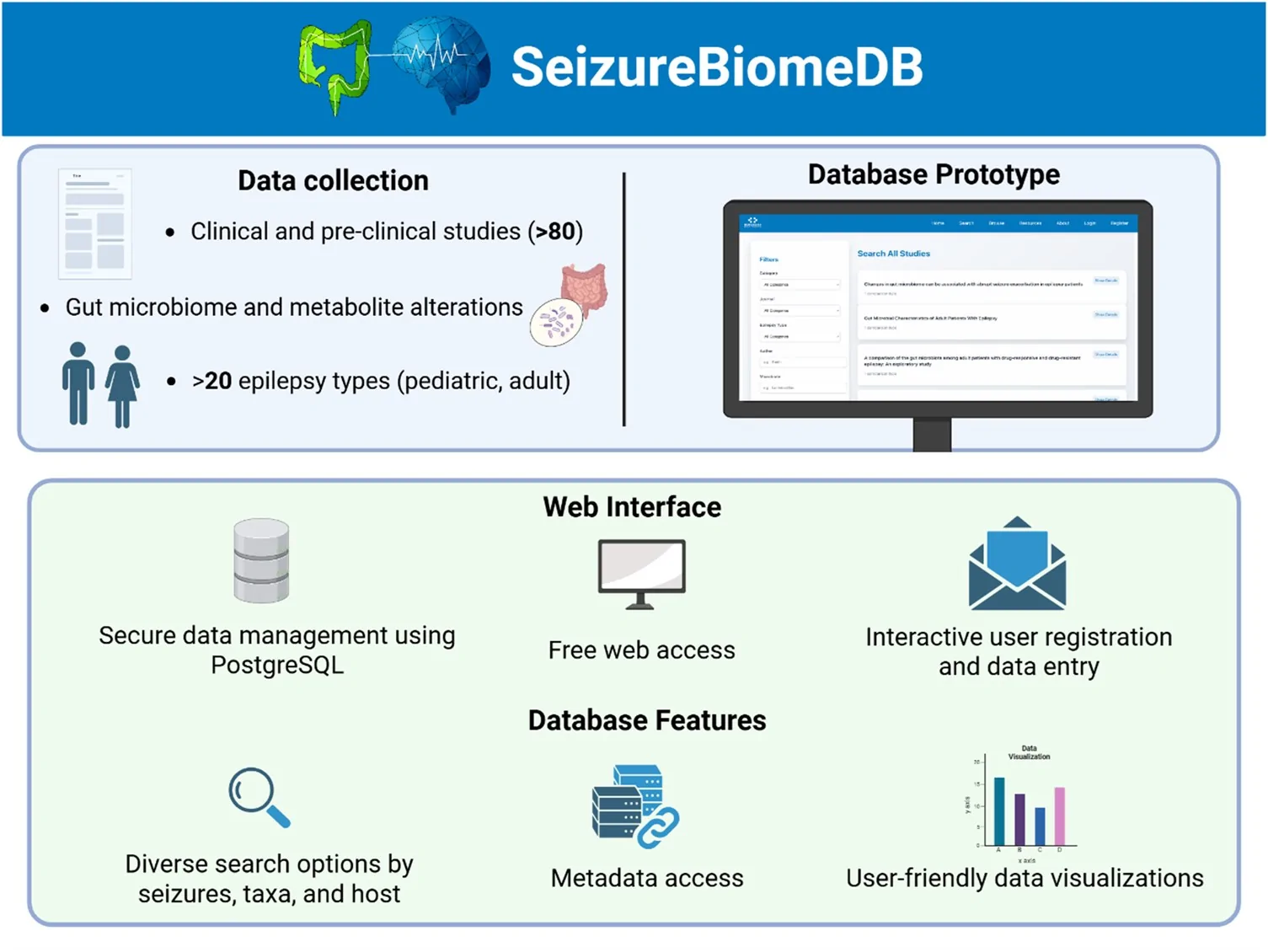

## 1 Introduction

Epilepsy is a neurological disorder characterized by recurrent, unprovoked seizures that affect approximately 50 million people worldwide (World Health Organization 2026). Based on the recommendation from the International League Against Epilepsy (Beniczky *et al.* 2025), seizures are classified into three major categories: focal, generalized, and unknown onset. Genetic, structural, and idiopathic factors have been known to cause epilepsy. However, recent studies have increasingly recognized the role of the gut microbiome as a potential environmental factor in epilepsy (Olson *et al*. 2018, Mu *et al*. 2022a, 2022b).

Gut microbiome refers to the diverse community of microorganisms within the gut, comprising bacteria, viruses, and fungi, as well as their genomic potential and functionalities (Rutsch *et al*. 2020). The gut microbiome communicates with the brain through the gut–brain axis, a bidirectional system of metabolic, immunological, endocrine, and neural signalling (Carabotti *et al*. 2015, Mu *et al*. 2016). One such pathway includes specific microbial metabolism that produce metabolites impacting the nervous system, such as gamma-aminobutyric acid (GABA) and short-chain fatty acids (SCFA) (Sharon *et al*. 2016, Dalile *et al*. 2019, Rutsch *et al*. 2020, Silva *et al*. 2020). A substantial body of research has demonstrated alterations in the gut microbiome among epilepsy patients, both before and after treatment by adrenocorticotropic hormone (ACTH) or the ketogenic diet (Xu *et al.* 2021).

Given the complexity of epilepsy etiology across developmental stages, findings on the gut microbiome vary widely across studies, making it challenging for researchers to capture the dynamic progress in this emerging field. Preexisting review articles provide a literature resource for this purpose (Dahlin and Prast-Nielsen 2019, Ravizza *et al.* 2025). Nevertheless, it remains challenging to discern how the gut microbiome changes in relation to different types of epilepsy and its treatments. To date, there is no centralized knowledge hub to integrate and navigate this rapidly developing area.

To help researchers better understand the microbiome in epilepsy, SeizureBiomeDB was created. By establishing an open-access database that aggregates current literature on the relationship between the gut microbiome and epilepsy, the database aims to create an interactive platform that integrates visualization tools and compiles data on demographics, epilepsy type, microbiome methodology, and taxonomic changes. Alterations of intestinal and circulating metabolites in relation to microbial metabolism are also integrated into the database. In the future, SeizureBiomeDB is designed to be a comprehensive and unique resource, making it easier for researchers to interpret findings in the interactions between the gut microbiome and epilepsy.

## 2 Methods

### 2.1 Literature search and data curation

**Figure 1:**
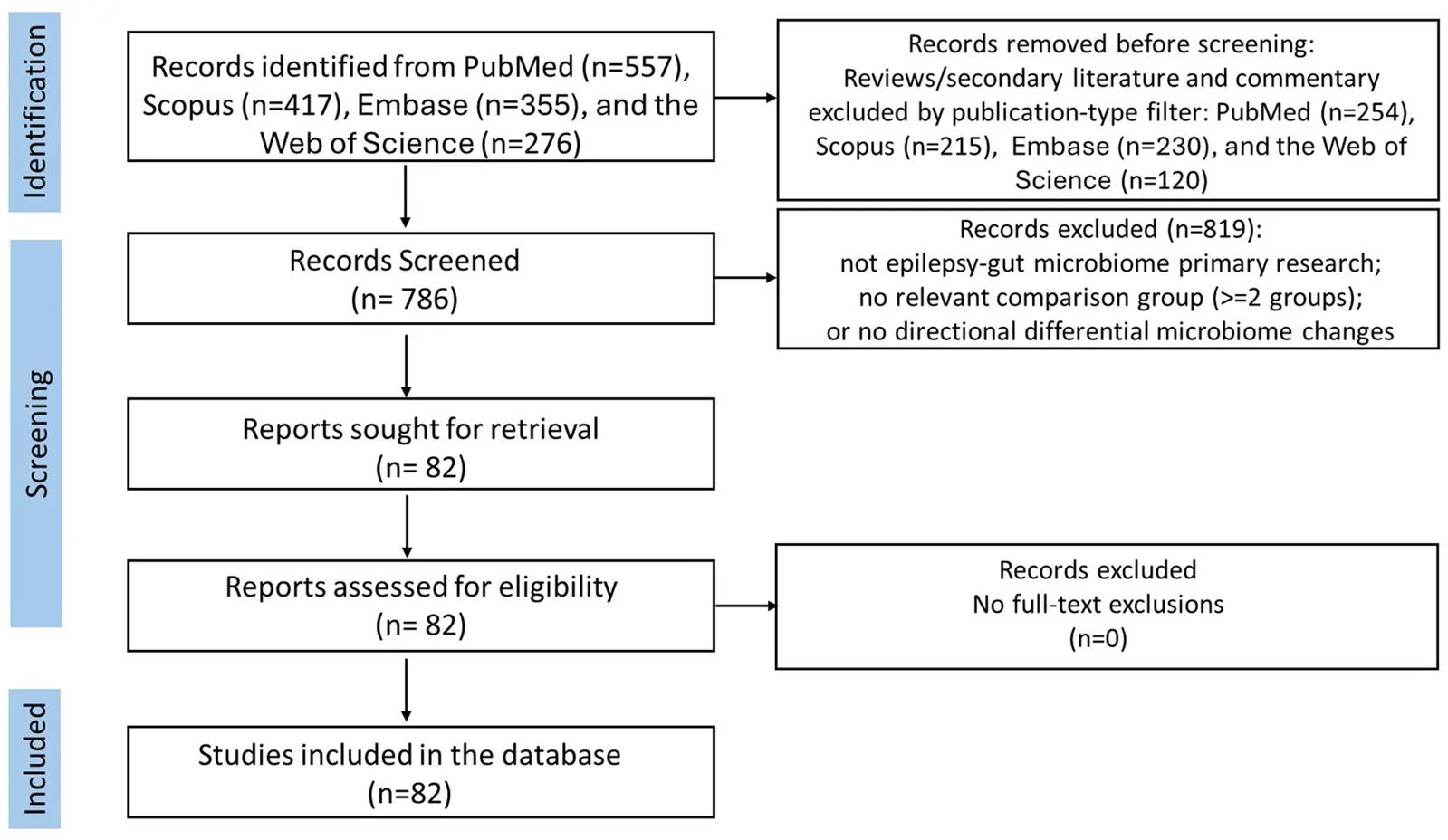
PRISMA flow diagram of study selection.

A literature search was conducted. The following search strategy was applied across PubMed, Scopus, Embase, and the Web of Science to identify relevant human and animal studies examining the relationship between epilepsy and the microbiome. The search query combined epilepsy-related terms with microbiome-related terms using Boolean operators as follows: (“epilepsy” OR “seizure” OR “seizures” OR “infantile spasm” OR “infantile spasms” OR “West syndrome” OR “Dravet syndrome” OR “status epilepticus” OR “drug-resistant epilepsy” OR “refractory epilepsy” OR “temporal lobe epilepsy” OR “focal epilepsy” OR “generalized epilepsy”) AND (“microbiome” OR “microbiota” OR “microbes” OR “gut microbiota” OR “gut microbiome” OR “intestinal microbiota” OR “intestinal microbiome” OR “gut flora” OR “dysbiosis” OR “gut-brain axis” OR “16S rRNA” OR “metagenomics” OR “probiotics” OR “prebiotics” OR “short-chain fatty acids” OR “Ketogenic diet” OR “SCFA” OR “Firmicutes” OR “Bacteroidetes” OR “Lactobacillus” OR “Bifidobacterium”). To capture both human and animal studies, no species filter was applied at the query level; instead, results were subsequently screened and categorized by study population (human or animal model) during the title and abstract review stage. In PubMed, the search was conducted using MeSH terms in combination with free-text keywords to maximize sensitivity: (“Epilepsy”[MeSH] OR “Seizures”[MeSH] OR “Spasms, Infantile”[MeSH] OR “epilepsy”[tiab] OR “seizure”[tiab] OR “infantile spasm”[tiab] OR “Dravet syndrome”[tiab]) AND (“Gastrointestinal Microbiome”[MeSH] OR “microbiome”[tiab] OR “microbiota”[tiab] OR “gut flora”[tiab] OR “dysbiosis”[tiab] OR “gut-brain axis”[tiab] OR “probiotics”[tiab]). The same conceptual structure was adapted for Scopus, Embase, and the Web of Science using their respective field tags (TITLE-ABS-KEY in Scopus; ti, ab, kw in Embase; TS = in Web of Science) to search titles, abstracts, and keywords. No language or date restrictions were applied during the initial search, and all database searches were conducted simultaneously to ensure consistency. Figure 1 summarizes the study identification and screening workflow (PRISMA flow diagram). Studies covered a diverse range of research subjects until 2025, including human, mice, rats, dogs, and Drosophila melanogaster. A list of literatures included in the study can be found in Supplementary document.

The inclusion criteria were: (i) published research articles exploring the relationship between the gut microbiome and epilepsy appearing in the abovementioned databases, (ii) reported changes in microbial taxa supported by significances and figures (e.g. bar plots, box plots, or other graphical representations showing differential abundance) or by tables listing taxa with reported changes between comparison groups, and (iii) inclusion of at least two comparison types (e.g. neurotypical versus epilepsy, control versus treatment). Studies reporting only descriptive microbiome composition without any visual or tabular representation of differential taxa were excluded. Both pre-clinical and clinical studies were included in this database. Data curation was performed independently by three authors (PD, AKT, CM), who extracted data from all included studies without knowledge of each other’s decisions and reached an initial agreement between the two curators >90%, reflecting a high level of concordance. Discrepancies were resolved through discussion and consensus and double-checked to ensure consistency with the literature. Curated information included study information (study type, sample type, host, epilepsy type, authors, journal, and year), participant information (age category, age, country, groups, and number of participants in each group), methodology (DNA extraction, sequencing platform, sequencing primers, sequencing region, raw sequencing data accession and link), and results (group comparisons, increased taxa/metabolites, decreased taxa/metabolites).

To ensure uniformity across all studies, taxonomic names are all mapped to the NCBI Taxonomy database to ensure consistency and unity. When nomenclatural changes have occurred (e.g. reclassification of genera), taxa were mapped to their current accepted names at the time of curation. When studies reported taxa at different taxonomic resolutions (e.g. one study at genus level, another at species level), both levels are preserved in the database to maintain fidelity to the original publications, with the taxonomic hierarchy enabling users to aggregate findings at their desired resolution level. Epilepsy types were standardized across all studies to facilitate the search process and enable structured comparisons across subtypes. The sample source, host, and sequencing methodology were also cross-referenced to ensure integrity.

### 2.2 Database architecture and interface development

SeizureBiomeDB interface was created using React.js (v18.3.1, Meta Platforms, Menlo Park, CA, USA) for the frontend, Express.js (v4.21.2, OpenJS Foundation, San Francisco, CA, USA) for the middleware, and PostgreSQL (v17.5, PostgreSQL Global Development Group, Berkeley, CA, USA) to manage the data, including encrypted login and password details. React.js was selected for its component-based architecture, which is well-suited to interactive data exploration interfaces; Express.js provides a lightweight, widely-supported middleware framework; and PostgreSQL offers robust relational data management with strong indexing capabilities for structured queries across studies, taxa, and metadata. A full security audit of all third-party dependencies was completed, with all 61 flagged items reviewed and confirmed to be contained within the build process. User authentication is handled via JSON Web Tokens (JWT), and passwords are stored using bcrypt hashing. SeizureBiomeDB does not store patient-level health data; all data are aggregated study-level findings from published literature. Following the completion of data curation within the Excel sheet, a custom Python (v3.11.5, Python Software Foundation, Wilmington, DE, USA) script was employed to format and insert the data into a PostgreSQL database table, designed with a specific schema. The script uses *pandas* (v2.2.3) to process data from the XLSX file. A backend API was built using Express.js to handle requests for study data. User authentication, user management, and administration are handled using JSON Web Tokens (JWT), allowing other researchers to access the data and add new studies. An administration approval feature has been implemented to ensure that user-added information is correct and up to date. To gain knowledge and understanding of the function and significance of microbes and metabolites, taxonomic names and metabolites are linked to external curated resources, such as List of Prokaryotic names with Standing in Nomenclature (LPSN, https://lpsn.dsmz.de/), MiMeDB (https://mimedb.org/) (Kruger *et al.* 2026), NCBI Taxonomy (https://www.ncbi.nlm.nih.gov/taxonomy), BacDive (https://bacdive.dsmz.de/) (Schober *et al.* 2025), KEGG (https://www.genome.jp/kegg/), and HMDB (https://www.hmdb.ca/) (Wishart *et al.* 2022). Knowledge databases that provide detailed introductions to specific taxa, such as PubMed, Microbiology Society Publications, Cell Press, Springer Nature, and Wiley Online Library, are also cross-referenced. A PDF export function has been embedded to allow users to retrieve data. Additionally, bulk data export in CSV format is available, allowing users to download curated datasets for independent re-analysis or meta-analysis. If the raw microbiome sequencing data is available in the studies, a link will also be provided to redirect to external resources, such as the European Nucleotide Archive and the NCBI Sequence Read Archive database. Quality control of raw sequencing data was performed by the original study authors as part of their published methods; SeizureBiomeDB curates the published findings and provides links to the original datasets for researchers who wish to conduct independent re-analysis. Direct DOI links to the original published articles are also provided for each curated study.

### 2.3 Design of search and visualization tools

To empower researchers with flexible access to the literature, a diverse range of visualization and search tools were designed. The search tools support text queries, as well as advanced filters such as author, journal, epilepsy subtype, age category (e.g. pediatric, adult, animal models), microbial taxa, and publication year. Furthermore, a Boolean toggle was integrated, utilizing AND/OR options to simplify the process of locating relevant literature. Search was built using a React component that retrieves study data from an API and applies client-side filtering with dynamic state management, Boolean logic, and grouped results, ensuring an efficient and user-friendly interface.

Visualization tools are also available to further enrich the database. A toggle underneath the *About the database* tab reveals a heat map showing the global study distribution of articles present. Taxonomic bar plots showing the direction of change are also included to illustrate microbiota changes on a study-by-study basis.

## 3 Results

### 3.1 Database overview

**Figure 2:**
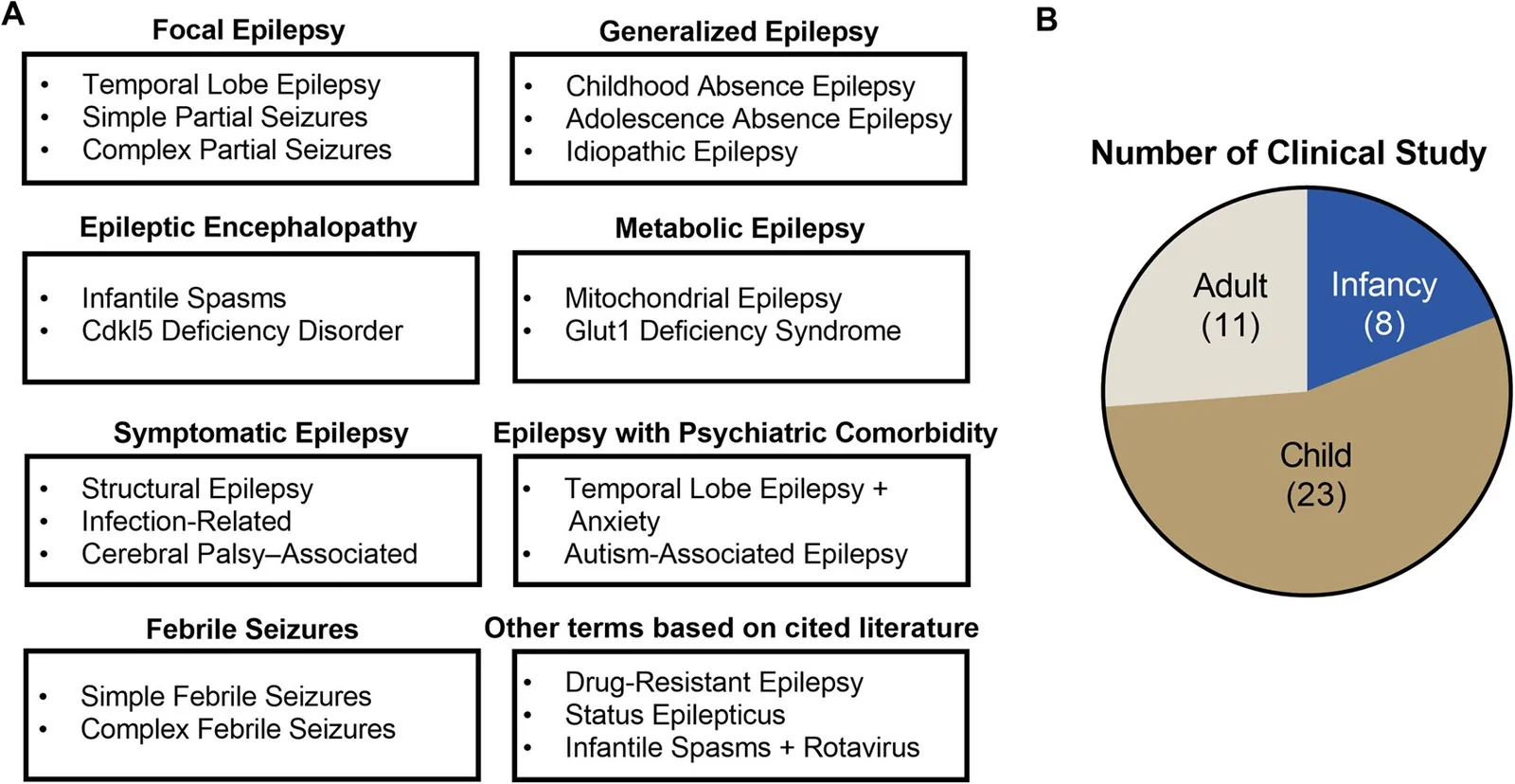
Distribution of epilepsy types across clinical studies in SeizureBiomeDB. (A) Epilepsy categories. Epilepsy types are labeled as reported in the original studies, illustrating the diversity of syndromes represented, including generalized, focal, drug-resistant, infantile spasms, febrile seizures, and mitochondrial epilepsy. (B) The number of studies by developmental stage: infancy (*n* = 19), childhood (*n* = 32), and adulthood (*n* = 15).

The study covered a diverse type of epilepsies considering the epilepsy type, etiology, and symptoms. These included focal epilepsy, generalized epilepsy, epileptic encephalopathy, metabolic epilepsy, symptomatic epilepsy, epilepsy with psychiatric comorbidity, and febrile seizures (Fig. 2A). All available clinical studies were included in the database, including infant studies, 12 childhood studies, and 11 adulthood studies (Fig. 2B).

**Figure 3:**
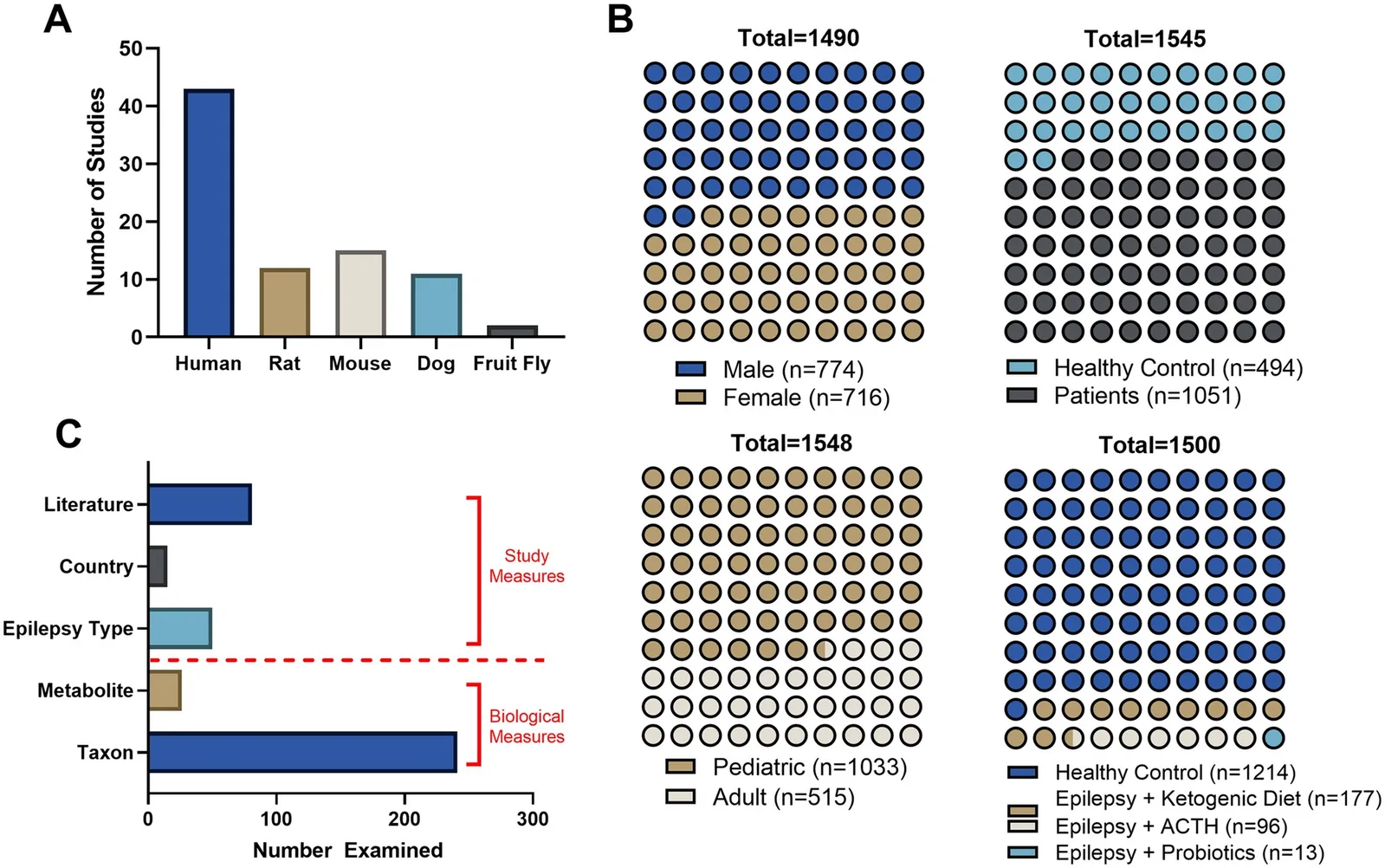
Study and participant characteristics in SeizureBiomeDB. (A) Distribution of studies by host species, with human studies predominating, followed by rodents, dogs, and fruit flies. (B) Participant demographics, including sex, patient versus control status, pediatric versus adult cohorts, and treatment subgroups (healthy controls, ketogenic diet, ACTH, probiotics). (C) Summary of study-level descriptors (literature source, country, epilepsy type) and biological measures (metabolites, taxa), highlighting the breadth of curated metadata.

The final database comprises over 80 studies, including clinical (pediatric, adult, ∼1500 participants) and pre-clinical studies using diverse animal models (e.g. mice, rats, dogs, *Drosophila melanogaster*, Fig. 3A and B). The database encompasses over 240 taxa, including bacterial and fungal populations, as well as 26 metabolites from host and microbial metabolism (Fig. 3C).

**Figure 4:**
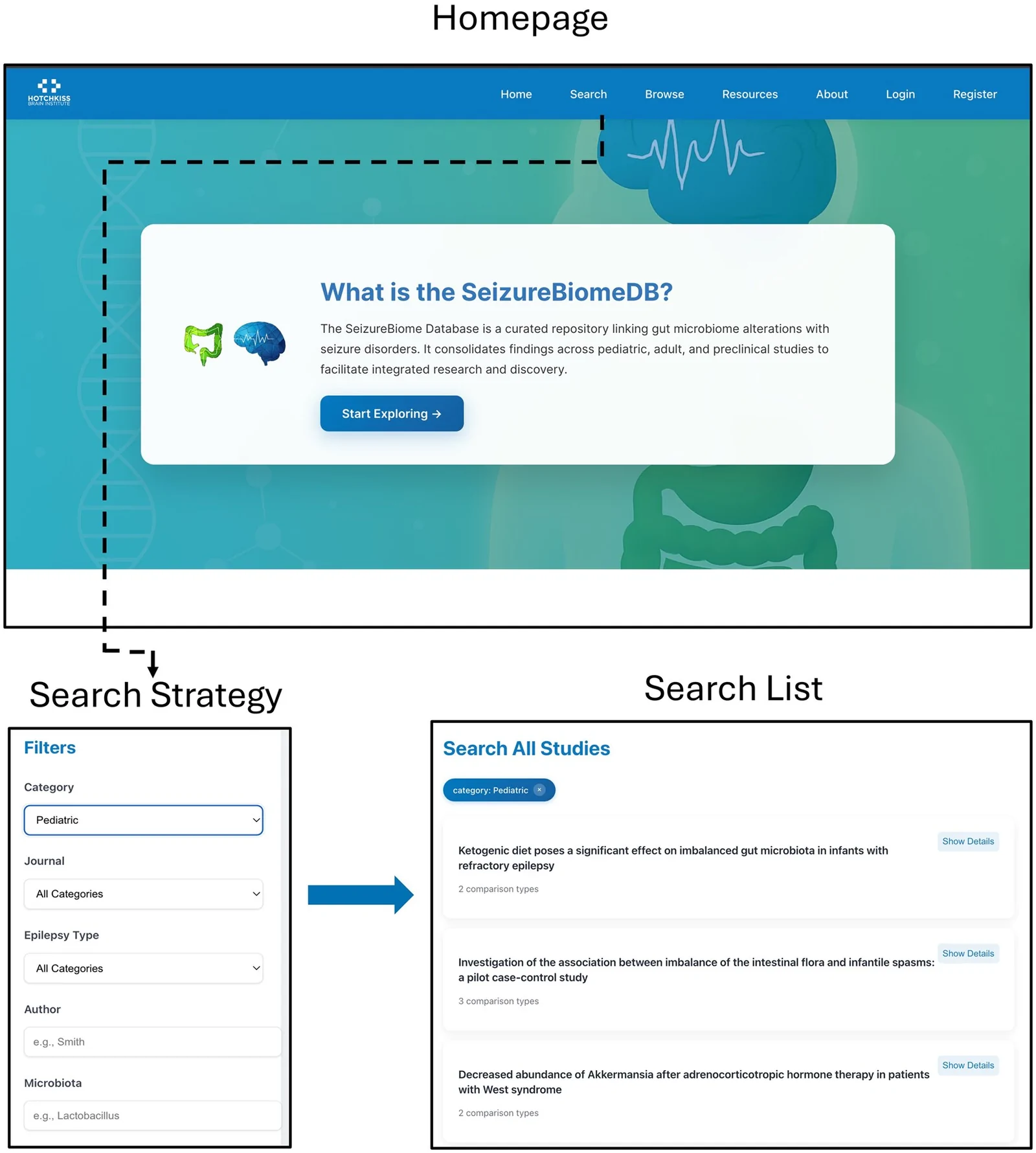
Navigation and search interface of SeizureBiomeDB. Screenshot of the homepage and search functions. Users can apply filters (e.g. category, journal, epilepsy type, author, microbial taxa) to retrieve study results. Search outputs preview study title, epilepsy type, comparison groups, and sample details, enabling streamlined exploration of epilepsy–microbiome literature.

When users browse the list of available studies, search results preview the author, epilepsy type, journal, sample size, year, and category before they access the article summary (Fig. 4). Underneath each study, a dropdown option allows users to view the comparison types listed in the survey.

### 3.2 Database access

**Figure 5:**
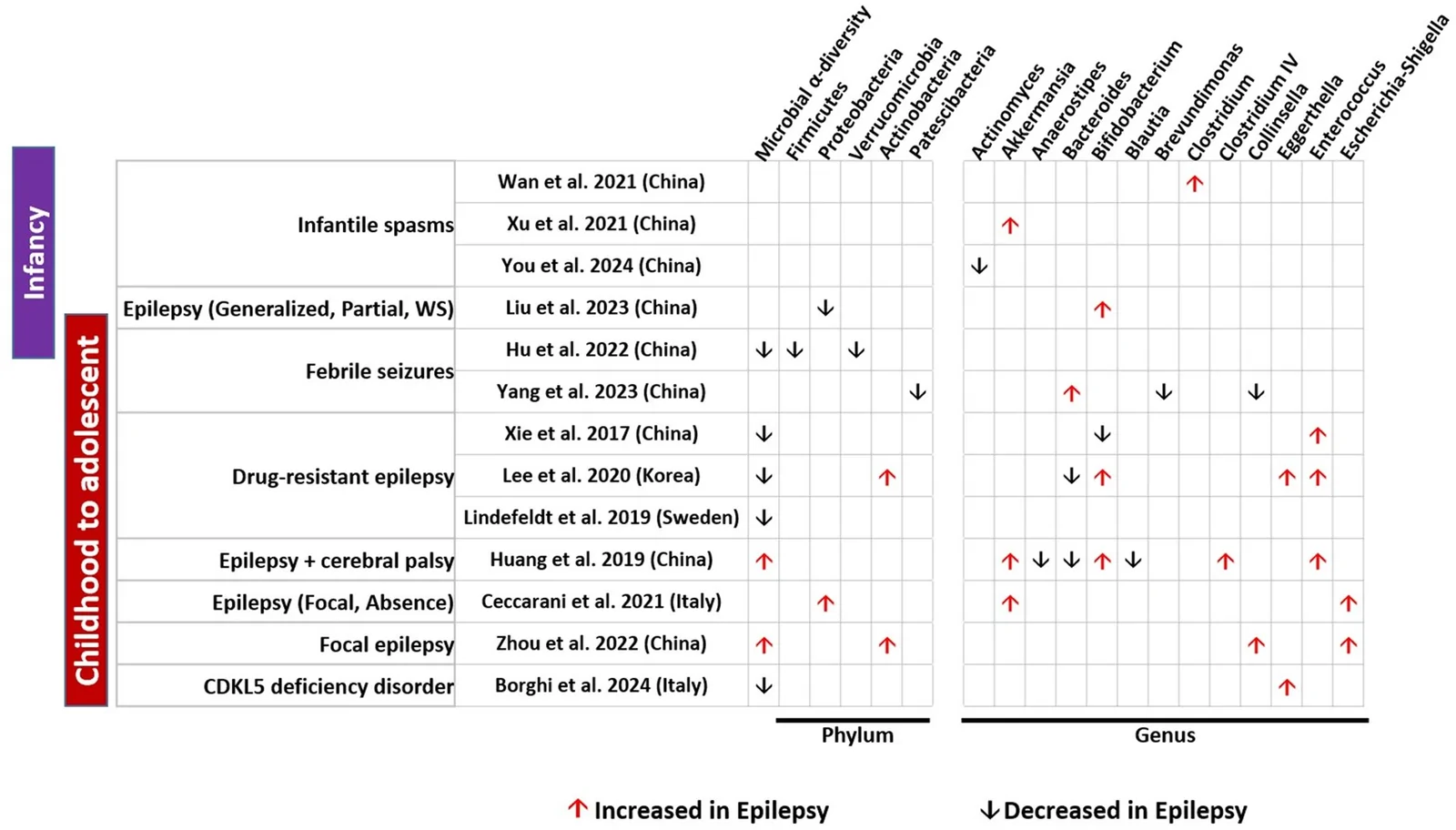
Phylum-level summary of gut microbiome alterations in pediatric epilepsy. Summary of reported phylum-level gut microbiome changes across pediatric epilepsy studies curated in SeizureBiomeDB, stratified by epilepsy subtype and developmental stage. Directional arrows indicate taxa reported as increased or decreased in epilepsy relative to control groups, as described in the original studies.

SeizureBiomeDB is publicly accessible at https://www.seizurebiome-db.ca/. This resource enables users to explore all existing findings on the gut microbiome and epilepsy through an intuitive interface. At the top of the database, there is a navigation bar with hyperlinks to a search tool accessible via the **Search** button or the **Browse** dropdown, which categorizes studies as either preclinical, adult, or pediatric (Fig. 5). Under each study title, it contains a menu with all available comparison types (Fig. 5). Once a user selects one, a breakdown of all relevant information is provided. The “Study Overview” column denotes study details, including study type, sample type, host, epilepsy type, authors, journal, and year, while the ‘Participant Info’ column specifies demographic and grouping information, including age, sex, country of origin, and group assignment (Fig. 5). The Methods section details the procedures for sample collection, including the data accession ID, DNA isolation kit, sequencing platform, primers, and targeted region. The Results section presents the comparison findings along with a list of taxa that increased or decreased.

The tab **Taxa Overview** on the left-hand side of the page reveals a taxonomic bar plot illustrating the change in microbiota composition as increased or decreased, visualized in a colour scheme (Fig. 5). Microbial alterations at the phylum, family, genus, and species levels, as well as changes in metabolites and microbial processes, where applicable, are presented.

SeizureBiomeDB includes a Resources page that offers information on microbial taxonomy (Genome Taxonomy Database, https://gtdb.ecogenomic.org/), microbial metabolite functions (MiMeDB, https://mimedb.org/), and epilepsy-related knowledge (Epilepsy Foundation, https://www.epilepsy.com/). A User Guide page is also provided on how to use SeizureBiomeDB. Additionally, it gives a **Submit** and **Registration** feature for researchers to contribute new findings on the gut microbiome and epilepsy. When users upload new information via the administration page, following the template, it will be reviewed, with the approval process managed by the administration before updates are made to the database.

### 3.3 Interactive heatmap

A major addition to SeizureBiomeDB is an interactive heatmap that integrates data across all studies in the database, covering microbial taxa and metabolites. The heatmap allows researchers to see at a glance which microbiome features are consistently reported as increased, decreased, or show mixed results across studies. It is filterable by organism type, epilepsy type, and direction of change. Users can search for specific taxa or filter by category, host organism, epilepsy type, or direction of change. Zoom controls allow users to compress the table for a broad overview or expand it for closer reading, and hovering over any cell shows the full details, including taxon name, study, and comparison. This tool supports cross-study taxon-level (family, class, order, family, genus, species) aggregation and comparison across epilepsy subtypes such as generalized versus focal epilepsy, going beyond what a conventional literature review can offer by providing an interactive, filterable, and visually integrated view of the data.

Taking the phylum and genus level for example, we generated a comparative summary highlighting key gut microbial taxa altered in pediatric epilepsy (Fig. 5). This summary integrates results across studies and visualizes directional changes, stratified by epilepsy subtype and developmental stage, thereby providing an overview of taxa that are commonly enriched or depleted in epilepsy. Across infancy, multiple studies reported reduced microbial alpha diversity alongside recurrent decreases in Firmicutes and Actinobacteria in infantile spasms and febrile seizures. In contrast, childhood and adolescent epilepsy subtypes demonstrated greater variability, with several studies reporting enrichment of Proteobacteria and Verrucomicrobia, particularly in focal and drug-resistant epilepsy. When comparing epilepsy subtypes, generalized epilepsy phenotypes more frequently exhibited reductions in commensal phyla associated with metabolic homeostasis, whereas focal epilepsy showed comparatively higher representation of potentially pro-inflammatory taxa. Together, this summary figure provides a high-level comparative framework for identifying microbial taxa that may represent common or distinct microbiome signatures across epilepsy syndromes.

### 3.4 Use case: ketogenic diet impact on gut microbiome in infants with refractory epilepsy

**Figure 6:**
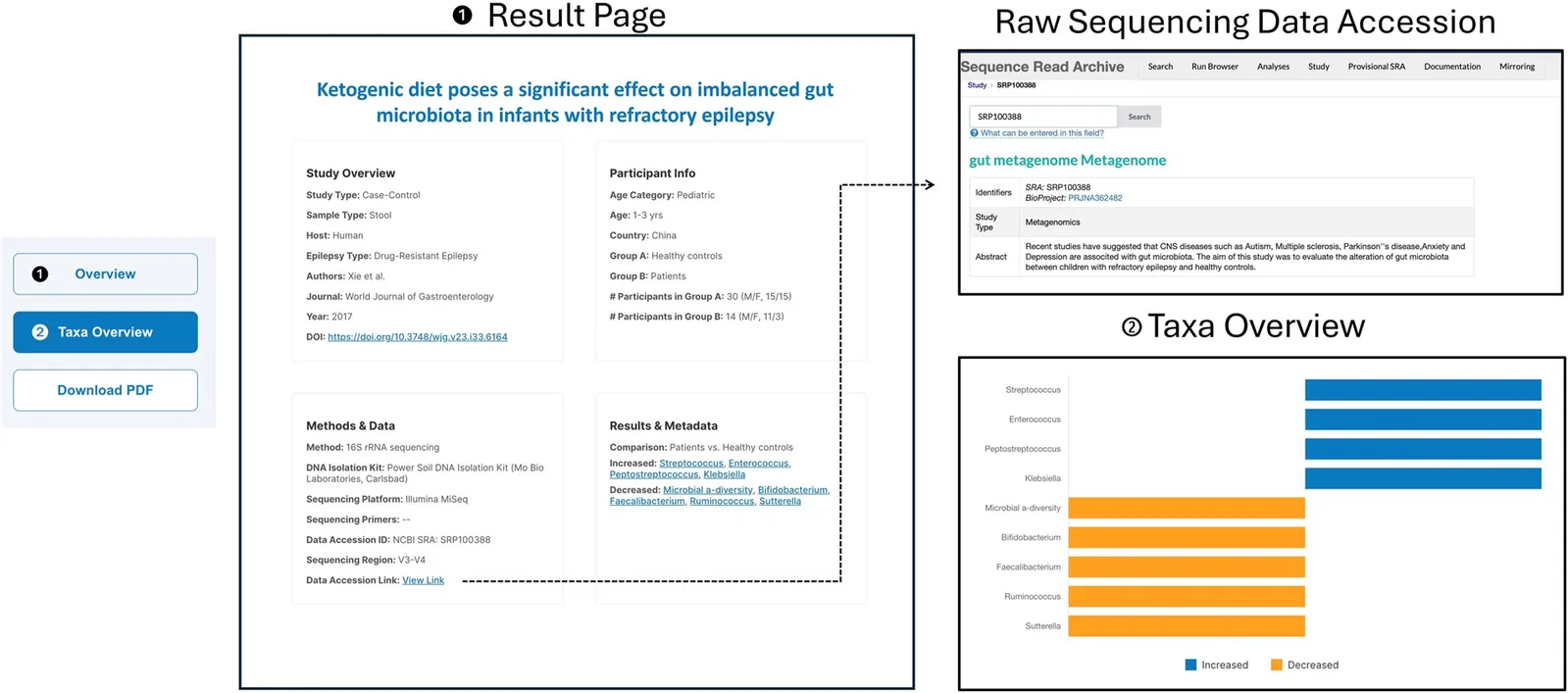
Study-level view and visualization tools in SeizureBiomeDB. Example result page showing metadata organization into Study Overview, Participant Info, Methods and Data, and Results and Metadata. Links to raw sequencing data are provided where available. Taxonomic bar plots visualize microbial changes (increased versus decreased taxa), supporting interpretation of study findings.

A researcher is interested in the effects of ketogenic diet therapy on the gut microbiome of infants with refractory epilepsy (Xie *et al.* 2017). To locate relevant studies using SeizureBiomeDB, the researcher can (i) navigate to the search page of SeizureBiomeDB, (ii) select “Drug-resistant Epilepsy” from the Epilepsy Type dropdown menu to filter for refractory epilepsy cases, (iii) select “Pediatric” from the Category dropdown menu to focus on infant- and child-related studies, (iv) enter “ketogenic” into the search bar to include ketogenic diet therapy as a key term, and (v) run the search to view results (Fig. 6). By applying these filters, the researcher is presented with most ketogenic-related studies in infants with refractory epilepsy.

## 4 Discussion

SeizureBiomeDB represents a comprehensive and specialized resource for epilepsy–microbiome research. By consolidating up-to-date published studies, it functions as an interactive systematic review and provides a unifying framework for a field often marked by fragmented and inconsistent findings. By integrating data across epilepsy subtypes, developmental stages, and methodologies, SeizureBiomeDB allows researchers to interpret microbiome and epilepsy findings in a curated centre hub.

The database offers practical tools to support comparative analysis. For example, some studies have reported reduced bacterial alpha diversity in drug-resistant epilepsy alongside shifts in taxa linked to short-chain fatty acid production, whereas others have observed no significant differences compared to healthy controls (Cui *et al.* 2022, Xu *et al.* 2021, He and Zhang 2024, Hong 2024). Interactive visualizations such as taxonomic bar plots and heatmaps help users navigate complex datasets, while direct links to sequencing accessions enhance transparency and reproducibility. Beyond descriptive associations, SeizureBiomeDB incorporates mechanistic insights by curating evidence that connects microbial changes to therapeutic interventions. Examples include the role of *Akkermansia muciniphila* and *Parabacteroides* in mediating ketogenic diet effects in animal models (Olson *et al.* 2018), or the shifts in microbial composition associated with ACTH treatment responsiveness in infantile spasms (Wan *et al.* 2024). This curated evidence connects reported microbial associations with therapeutic contexts, highlighting potential avenues for mechanistic investigation and underscoring the potential of microbiome features as biomarkers or therapeutic targets.

SeizureBiomeDB is designed as a living, community-driven platform. Its standardized metadata and taxonomy enable structured comparisons across studies, while its submission system allows researchers to contribute new findings, ensuring the database remains current as the field evolves. Taken together, these features distinguish SeizureBiomeDB not merely as a catalog of microbiome–epilepsy studies, but as a dynamic resource that supports ongoing discovery, and lays the groundwork for future translational advances.

Several databases, such as Disbiome (Janssens *et al.* 2018) and gutMDisorder (Qi *et al.* 2023), have cataloged microbiome–disease associations among a broad disease context. These resources provide valuable overviews on the gut microbiome in health and disease. In the present study, SeizureBiomeDB is tailored to epilepsy and offers a disease-specific, comprehensive, and interactive platform dedicated exclusively to epilepsy and the gut microbiome. It consolidates all available epilepsy-focused microbiome studies, systematically integrates findings across epilepsy subtypes (focal epilepsy, generalized epilepsy, infantile epileptic spasms syndrome, febrile seizures, etc.), and provides direct links to data accessions. Furthermore, it uniquely incorporates information on therapeutic interventions such as ketogenic diet and ACTH treatment that allow researchers to understand the impact of intervention on gut microbiome. Other features, including raw data accession and community-driven tools, will further allow researchers to collectively contribute to the rapidly growing field.

###  4.1 Future developments

As SeizureBiomeDB continues to develop and evolve, several enhancements and new features are planned to increase its utility for researchers. One of the primary goals is to introduce user-driven annotation tools to enable insights beyond what is solely listed in the paper. A tiered user access model will be incorporated to distinguish between verified contributors and casual users, ensuring data integrity. Furthermore, future versions aim to support Application Programming Interface access for other developers who want to use this tool. Another key objective of the platform is to incorporate multi-omics datasets, including metabolomics and proteomics (Wang *et al.* 2025). This integration aims to facilitate a more comprehensive analysis, potentially yielding new insights and fostering a deeper understanding of the microbiome–gut–brain axis and epilepsy. Ultimately, we strive to automate the data extraction process moving forward. Relying on community members to continue the literature review may leave gaps in the database curation. Through natural language processing and an AI-assisted pipeline, manual effort and inconsistencies can be significantly reduced, making updates more efficient. Additional planned features include the ability for users to save selected studies, export curated subsets, and retain search history to support reproducible literature review workflows. Interoperability with existing databases through standardized data export formats will also be pursued to promote integration with broader microbiome resources.

## 5 Conclusion

The gut microbiome plays a crucial role in regulating neurological issues such as epilepsy, making a comprehensive exploration of this topic essential. In this database, all studies are indexed and formatted, ensuring information is easily accessible to everyone. Data visualization features, including global study distributions and taxonomic bar plots, have enriched this dataset. SeizureBiomeDB has been launched, featuring over 80 studies and 240 identified taxa. This resource will continue to be updated, featuring tools designed to enhance our understanding of the microbiome–gut–brain axis and its role in epilepsy.

## Supplementary Material

vbag198_Supplementary_Data

## Data Availability

The database can be accessed through https://www.seizurebiome-db.ca/. The source code is publicly available on GitHub at https://github.com/AD-txigfwbexxk23/SeizureBiomeDB.
