# Supplementary material for: SeizureBiomeDB: a unified database of gut microbiome alterations in pediatric and adult epilepsy
Source: Bioinform Adv. 2026 Aug 6;6(1):vbag198. doi: 10.1093/bioadv/vbag198 (PMC13501535; doi:10.1093/bioadv/vbag198)
Supplement: Supplementary file 1 [file vbag198_supplementary_data.pdf]

SUPPLEMENTAL DOCUMENTS

Subject Section

SeizureBiomeDB: A unified database of gut microbiome alterations in pediatric and adult epilepsy

Zehaan Walji[1,2], Parshva Dave[1,2], Meherab Ali[3,5], Omar Negmeldin[3,5], Armaan Kaur Toor[4], Jane Shearer[5,6,7], Chunlong Mu[4,5,6,7]*

[1] Department of Computer Science, University of Calgary, Calgary, Alberta, T2N 1N4, Canada
[2] Department of Biological Science, University of Calgary, Calgary, Alberta, T2N 1N4, Canada
[3] Department of Neuroscience, University of Calgary, Calgary, Alberta, T2N 1N4, Canada
[4] Department of Microbiology, Immunology, and Infectious Diseases, Cumming School of Medicine, University of Calgary, Calgary, AB T2N 1N4, Canada
[5]Faculty of Kinesiology & Department of Biochemistry and Molecular Biology, Cumming School of Medicine, University of Calgary, Calgary, Alberta, T2N 1N4, Canada
[6] Hotchkiss Brain Institute, Cumming School of Medicine, University of Calgary, Calgary, AN, T2N 4N1, Canada
[7]Alberta Children's Hospital Research Institute, University of Calgary, Calgary, Alberta, Canada

*Correspondence. Department of Microbiology, Immunology, and Infectious Diseases, Cumming School of Medicine, University of Calgary, chunlong.mu1@ucalgary.ca

This document lists all literatures used for developing the database. Studies covered a diverse range of research subjects until 2025, including human (pediatrics(Tagliabue *et al.* 2017, Tevzadze, Shanshiashvilli, and Mikeladze 2017, Xie *et al.* 2017, Zhang *et al.* 2018, Huang *et al.* 2019, Lindefeldt *et al.* 2019, Żarnowska *et al.* 2019, Lee K *et al.* 2020, Ceccarani *et al.* 2021, Ferraris *et al.* 2021, Wan *et al.* 2021, 2024, Xu *et al.* 2021, Zeng and Hu 2021, Bertuccioli *et al.* 2022, Dahlin M *et al.* 2022, Dai *et al.* 2022, Zhou C *et al.* 2022, Hu *et al.* 2023, Liu *et al.* 2023, Lum *et al.* 2023, Peng *et al.* 2023, Wang *et al.* 2023, Yang and Tian 2023, Borghi *et al.* 2024, Dahlin Maria, Wheelock, and Prast-Nielsen 2024, You *et al.* 2024, Chojnowski *et al.* 2025, Fang *et al.* 2025, Freibauer, Pai, and RamachandranNair 2025, Mohamed *et al.* 2025, Riva *et al.* 2025, Steriade *et al.* 2025, Yuwattana *et al.* 2025), adults(Gong Xue *et al.* 2020, Lee H *et al.* 2021, Cui *et al.* 2022, Dong *et al.* 2022, Wei *et al.* 2023, Zhou K *et al.* 2023, Kwack *et al.* 2024)), mice(Olson *et al.* 2018, Caro De *et al.* 2019, Eor *et al.* 2021, Miljanovic and Potschka 2021, Ding *et al.* 2022, Lum *et al.* 2023, Kebede *et al.* 2024, Kim *et al.* 2024, Li Xinyu *et al.* 2024, Mu C *et al.* 2024, Wlaź *et al.* 2024, Wu *et al.* 2024, Zierath *et al.* 2024, Özcan *et al.* 2025, Yu *et al.* 2025), rats(Citraro *et al.* 2020, Gong Xiaoxiang *et al.* 2022, Lima de *et al.* 2022, Li Xiang *et al.* 2022, Medel-Matus *et al.* 2022, Medel-Matus *et al.* 2022, Mu C *et al.* 2022, Mu Chunlong, Nikpoor *et al.* 2022, Mu Chunlong, Pochakom *et al.* 2022, Oliveira *et al.* 2022, Thai *et al.* 2023, Li B *et al.* 2024, Riva *et al.* 2024, Chen *et al.* 2025), dogs(PA Lansdon 2018, Muñana, Jacob, and Callahan 2020, Pilla *et al.* 2020, García-Belenguer *et al.* 2021, Ledeganck *et al.* 2022, Watanangura *et al.* 2022, 2024, Garcia-Belenguer *et al.* 2023, Verdoodt *et al.* 2024, 2025, Aksoy, Yaman, and Çiftci 2025, Silvestrino *et al.* 2025), and Drosophila melanogaster(Lansdon, Kasuya, and Kitamoto 2024).

References

Aksoy N, Yaman Ş, Çiftci A. Comparative assessment of faecal microbiota in healthy and epileptic dogs. *Journal of Anatolian Environmental and Animal Sciences* 2025;**10**(1):79–86.

Bertuccioli A, Cardinali M, Pierro F Di *et al.* Ketogenic and Low FODMAP Diet in Therapeutic Management of a Young Autistic Patient with Epilepsy and Dysmetabolism Poorly Responsive

to Therapies: Clinical Response and Effects of Intestinal Microbiota. *Int J Mol Sci* 2022;**23**(15). https://doi.org/Artn 8829 10.3390/Ijms23158829.

Borghi E, Xynomilakis O, Ottaviano E *et al.* Gut microbiota profile in CDKL5 deficiency disorder patients. *Sci Rep* 2024;**14**(1):7376.

Caro C De, Leo A, Nesci V *et al.* Intestinal inflammation increases convulsant activity and reduces antiepileptic drug efficacy in a mouse model of epilepsy. *Sci Rep* 2019;**9**(1):13983.

Ceccarani C, Viganò I, Ottaviano E *et al.* Is gut microbiota a key player in epilepsy onset? A longitudinal study in drug-naive children. *Front Cell Infect Microbiol* 2021;**11**:749509.

Chen L, Li J, Zhang W *et al.* Antiepileptic Effects of Acorus tatarinowii Schott in a Rat Model of Epilepsy: Regulation of Metabolic Axes and Gut Microbiota. *Biology (Basel)* 2025;**14**(5):488.

Chojnowski K, Opiełka M, Urbanowicz K *et al.* Untargeted metabolomics reveals key metabolic alterations in pediatric epilepsy with insights into Tryptophan metabolism and the gut–brain axis. *Sci Rep* 2025;**15**(1):15262.

Citraro R, Lembo F, Caro C De *et al.* First evidence of an altered microbiota and intestinal damage and its link to absence epilepsy in a genetic animal model, the WAG/Rij rat. *Epilepsia* 2020.

Cui G, Liu S, Liu Z *et al.* Gut microbiome distinguishes patients with epilepsy from healthy individuals. *Front Microbiol* 2022;**12**:696632.

Dahlin M, Singleton SS, David JA *et al.* Higher levels of Bifidobacteria and tumor necrosis factor in children with drug-resistant epilepsy are associated with anti-seizure response to the ketogenic diet. *EBioMedicine* 2022;**80**. https://doi.org/Artn 104061 10.1016/J.Ebiom.2022.104061.

Dahlin Maria, Wheelock CE, Prast-Nielsen S. Association between seizure reduction during ketogenic diet treatment of epilepsy and changes in circulatory metabolites and gut microbiota composition. *EBioMedicine* 2024;**109**.

Dai Y, Wang M, Zhong D *et al.* Bacillus subtilis plays a role in the inhibition of transporter ABCB1 in Caco-2 cells. *Epilepsy Res* 2022;**183**:106925.

Ding X, Zhou J, Zhao L *et al.* Intestinal flora composition determines microglia activation and improves epileptic episode progress. *Front Cell Infect Microbiol* 2022;**12**:835217.

Dong L, Zheng Q, Cheng Y *et al.* Gut microbial characteristics of adult patients with epilepsy. *Front Neurosci* 2022;**16**:803538.

Eor JY, Tan PL, Son YJ *et al.* Gut microbiota modulation by both Lactobacillus fermentum MSK 408 and ketogenic diet in a murine model of pentylenetetrazole-induced acute seizure. *Epilepsy Res* 2021;**169**:106506.

Fang S, Hu N, Zhou C *et al.* The comparison of gut microbiota between different types of epilepsy in children. *Microb Cell Fact* 2025;**24**(1):64.

Ferraris C, Meroni E, Casiraghi MC *et al.* One Month of Classic Therapeutic Ketogenic Diet Decreases Short Chain Fatty Acids Production in Epileptic Patients. *Front Nutr* 2021;**8**. https://doi.org/Artn 613100 10.3389/Fnut.2021.613100.

Freibauer A, Pai N, RamachandranNair R. Characterizing the fecal microbiome in patients on the ketogenic diet for drug resistant epilepsy. *Heliyon* 2025;**11**(1).

Garcia-Belenguer S, Grasa L, Palacio J *et al.* Effect of a Ketogenic Medium Chain Triglyceride-Enriched Diet on the Fecal Microbiota in Canine Idiopathic Epilepsy: A Pilot Study. *Vet Sci* 2023;**10**(4). https://doi.org/10.3390/vetsci10040245.

García-Belenguer S, Grasa L, Valero O *et al.* Gut microbiota in canine idiopathic epilepsy: effects of disease and treatment. *Animals* 2021;**11**(11):3121.

Gong Xiaoxiang, Liu L, Li X *et al.* Neuroprotection of cannabidiol in epileptic rats: Gut microbiome and metabolome sequencing. *Front Nutr* 2022;**9**:1028459.

Gong Xue, Liu XU, Chen C *et al.* Alteration of gut microbiota in patients with epilepsy and the potential index as a biomarker. *Front Microbiol* 2020;**11**:517797.

Hu B, Zhu X, Zheng J *et al.* Changes of serum metabolomics and gut microbiota reveal specific characteristics of children with febrile seizures. *Eur J Neurol* 2023;**30**(11):3516–28.

Huang C, Li Y, Feng X *et al.* Distinct gut microbiota composition and functional category in children with cerebral palsy and epilepsy. *Front Pediatr* 2019;**7**:394.

Kebede V, Ravizza T, Balosso S *et al.* Early treatment with rifaximin during epileptogenesis reverses gut alterations and reduces seizure duration in a mouse model of acquired epilepsy. *Brain Behav Immun* 2024;**119**:363–80.

Kim Y, Jang Y, Shin YW *et al.* Gut microbiome diversity in a febrile seizure mouse model. *Encephalitis* 2024;**4**(1):11.

Kwack DW, Lee S, Lee DH *et al.* Changes in gut microbiome can be associated with abrupt seizure exacerbation in epilepsy patients. *Clin Neurol Neurosurg* 2024;**246**:108556.

Lansdon P, Kasuya J, Kitamoto T. Commensal bacteria exacerbate seizure-like phenotypes in Drosophila voltage-gated sodium channel mutants. *Genes Brain Behav* 2024;**23**(5):e70000.

Ledeganck L, Verdoodt F, Hesta M *et al.* The gut-brain axis: Effect of antibiotics on canine drug-resistant idiopathic epilepsy. *Vlaams Diergeneeskd Tijdschr* 2022;**91**(6).

Lee H, Lee S, Lee DH *et al.* A comparison of the gut microbiota among adult patients with drug-responsive and drug-resistant epilepsy: an exploratory study. *Epilepsy Res* 2021;**172**:106601.

Lee K, Kim N, Shim JO *et al.* Gut bacterial dysbiosis in children with intractable epilepsy. *J Clin Med* 2020;**10**(1):5.

Li B, Ma Y, Wang X *et al.* Ketogenic Diets Alter the Gut Microbiome, Resulting in Decreased Susceptibility to and Cognitive Impairment in Rats with Pilocarpine-Induced Status Epilepticus. *Neurochem Res* 2024;**49**(10):2726–42.

Li Xiang, Wang Q, Wu D *et al.* The effect of a novel anticonvulsant chemical Q808 on gut microbiota and hippocampus neurotransmitters in pentylenetetrazole-induced seizures in rats. *BMC Neurosci* 2022;**23**(1):7.

Li Xinyu, Li J, Ji J *et al.* Gut microbiota modification by diosgenin mediates antiepileptic effects in a mouse model of epilepsy. *J Neurochem* 2024;**168**(12):3982–4000.

Lima AMDL de, Lima Rosa G de, Guzzo EFM *et al.* Gut microbiota modulation by prednisolone in a rat kindling model of pentylenetetrazol (PTZ)-induced seizure. *Microb Pathog* 2022;**163**:105376.

Lindefeldt M, Eng A, Darban H *et al.* The ketogenic diet influences taxonomic and functional composition of the gut microbiota in children with severe epilepsy. *NPJ Biofilms Microbiomes* 2019;**5**. https://doi.org/Unsp 5 10.1038/S41522-018-0073-2.

Liu T, Jia F, Guo Y *et al.* Altered intestinal microbiota composition with epilepsy and concomitant diarrhea and potential indicator biomarkers in infants. *Front Microbiol* 2023;**13**:1081591.

Lum GR, Ha SM, Olson CA *et al.* Ketogenic diet therapy for pediatric epilepsy is associated with alterations in the human gut microbiome that confer seizure resistance in mice. *Cell Rep* 2023;**42**(12).

Medel-Matus J, Lagishetty V, Santana-Gomez C *et al.* Susceptibility to epilepsy after traumatic brain injury is associated with preexistent gut microbiome profile. *Epilepsia* 2022;**63**(7):1835–48.

Medel-Matus JS, Simpson CA, Ahdoot AI *et al.* Modification of post-traumatic epilepsy by fecal microbiota transfer. *Epilepsy & Behavior* 2022;**134**:108860.

Miljanovic N, Potschka H. The impact of Scn1a deficiency and ketogenic diet on the intestinal microbiome: A study in a genetic Dravet mouse model. *Epilepsy Res* 2021;**178**. https://doi.org/ARTN 106826 10.1016/j.eplepsyres.2021.106826.

Mohamed MA, Mahmoud EA, Basily MS *et al.* Efficacy of treating Helicobacter pylori infection on seizure frequency in children with drug-resistant idiopathic generalized epilepsy: a randomized controlled trial. *Ital J Pediatr* 2025;**51**(1):121.

Mu C, Choudhary A, Mayengbam S *et al.* Seizure modulation by the gut microbiota and tryptophan-kynurenine metabolism in an animal model of infantile spasms. *EBioMedicine* 2022;**76**:103833. https://doi.org/10.1016/j.ebiom.2022.103833.

Mu C, Kesler M, Chen X *et al.* Exogenous ketones exert antiseizure effects and modulate the gut microbiome and mycobiome in a clinically relevant murine model of epilepsy. *Epilepsia* published online 2024. https://doi.org/10.1111/epi.18150.

Mu Chunlong, Nikpoor N, Tompkins TA *et al.* Targeted gut microbiota manipulation attenuates seizures in a model of infantile spasms syndrome. *JCI Insight* 2022;**7**(12).

Mu Chunlong, Pochakom A, Reimer RA *et al.* Addition of prebiotics to the ketogenic diet improves metabolic profile but does not affect seizures in a rodent model of infantile spasms syndrome. *Nutrients* 2022;**14**(11):2210.

Muñana KR, Jacob ME, Callahan BJ. Evaluation of fecal Lactobacillus populations in dogs with idiopathic epilepsy: a pilot study. *Anim Microbiome* 2020;**2**(1):19.

Oliveira MET, Paulino GVB, Santos Júnior ED dos *et al.* Multi-omic analysis of the gut microbiome in rats with lithium-pilocarpine-induced temporal lobe epilepsy. *Mol Neurobiol* 2022;**59**(10):6429–46.

Olson CA, Vuong HE, Yano JM *et al.* The gut microbiota mediates the anti-seizure effects of the ketogenic diet. *Cell* 2018;**173**(7):1728–41.

Özcan E, Yu KB, Dinh L *et al.* Dietary fiber content in clinical ketogenic diets modifies the gut microbiome and seizure resistance in mice. *Nat Commun* 2025;**16**(1):987.

PA Lansdon. Bidirectional communication between the brain and gut microbiota in Shudderer, a Drosophila Nav channel mutant. Iowa , 2018.

Peng Y, Chiu ATG, Li VWY *et al.* The role of the gut-microbiome-brain axis in metabolic remodeling amongst children with cerebral palsy and epilepsy. *Front Neurol* 2023;**14**:1109469.

Pilla R, Law TH, Pan Y *et al.* The Effects of a Ketogenic Medium-Chain Triglyceride Diet on the Feces in Dogs With Idiopathic Epilepsy. *Front Vet Sci* 2020;**7**:541547. https://doi.org/10.3389/fvets.2020.541547.

Riva A, Sahin E, Volpedo G *et al.* Identification of an epilepsy-linked gut microbiota signature in a pediatric rat model of acquired epilepsy. *Neurobiol Dis* 2024;**194**:106469.

Riva A, Sahin E, Volpedo G *et al.* Medication-resistant epilepsy is associated with a unique gut microbiota signature. *Epilepsia* 2025;**66**(7):2268–84.

Silvestrino M, Pirolo M, Bianco A *et al.* Idiopathic epilepsy in dogs is associated with dysbiotic faecal microbiota. *Anim Microbiome* 2025;**7**(1):31.

Steriade C, Thomas SC, Xu F *et al.* Patients with status epilepticus and new-onset refractory status epilepticus display drastically altered fecal microbiomes compared to chronic epilepsy patients. *Epilepsia* 2025;**66**(9):3254–68.

Tagliabue A, Ferraris C, Uggeri F *et al.* Short-term impact of a classical ketogenic diet on gut microbiota in GLUT1 Deficiency Syndrome: A 3-month prospective observational study. *Clin Nutr ESPEN* 2017;**17**:33–7. https://doi.org/10.1016/j.clnesp.2016.11.003.

Tevzadze G, Shanshiashvilli L, Mikeladze D. Children with epilepsy and autistic spectrum disorders show similarly high levels of urinary p-cresol. *J Biol Phys Chem* 2017;**17**(2):77–80.

Thai K, Taylor MW, Fernandes T *et al.* Topiramate alters the gut microbiome to aid in its anti-seizure effect. *Front Microbiol* 2023;**14**:1242856.

Verdoodt F, Hemeryck L, Molina J *et al.* The idiopathic epilepsy mystery: faecal metabolome and microbiome are altered in dogs with idiopathic epilepsy. *Benelux Metabolomics Days 2024* 2024.

Verdoodt F, Hesta M, Goossens E *et al.* The fecal metabolome and microbiome are altered in dogs with idiopathic epilepsy compared to healthy dogs. *Sci Rep* 2025;**15**(1):27024.

Wan L, Shi X, Yan H *et al.* Abnormalities in Clostridioides and related metabolites before ACTH treatment may be associated with its efficacy in patients with infantile epileptic spasm syndrome. *CNS Neurosci Ther* 2024;**30**(1):e14398.

Wan L, Yang G, Zhang S *et al.* Investigation of the association between imbalance of the intestinal flora and infantile spasms: a pilot case-control study. *Transl Pediatr* 2021;**10**(4):819.

Wang J, Huang Li, Li H *et al.* Effects of ketogenic diet on the classification and functional composition of intestinal flora in children with mitochondrial epilepsy. *Front Neurol* 2023;**14**:1237255.

Watanangura A, Meller S, Farhat N *et al.* Behavioral comorbidities treatment by fecal microbiota transplantation in canine epilepsy: A pilot study of a novel therapeutic approach. *Front Vet Sci* 2024;**11**:1385469.

Watanangura A, Meller S, Suchodolski JS *et al.* The effect of phenobarbital treatment on behavioral comorbidities and on the composition and function of the fecal microbiome in dogs with idiopathic epilepsy. *Front Vet Sci* 2022;**9**:933905.

Wei S, Mai Y, Hu L *et al.* Altered gut microbiota in temporal lobe epilepsy with anxiety disorders. *Front Microbiol* 2023;**14**:1165787.

Wlaź P, Wiater A, Majewska M *et al.* Effect of dietary supplementation with Lactobacillus helveticus R0052 on seizure thresholds and antiseizure potency of sodium valproate in mice. *Psychopharmacology (Berl)* 2024;**241**(2):327–40.

Wu Y, Gu D, Li J *et al.* Role of the gut microbiota in cefoperazone/sulbactam-induced epilepsy in mice with chronic renal failure. *Ren Fail* 2024;**46**(2):2371551.

Xie G, Zhou Q, Qiu CZ *et al.* Ketogenic diet poses a significant effect on imbalanced gut microbiota in infants with refractory epilepsy. *World J Gastroenterol* 2017;**23**(33):6164–71. https://doi.org/10.3748/wjg.v23.i33.6164.

Xu L, Chen D, Zhao C *et al.* Decreased abundance of Akkermansia after adrenocorticotropic hormone therapy in patients with West syndrome. *BMC Microbiol* 2021;**21**(1):126.

Yang L, Tian J. Changes of intestinal flora in children with febrile seizure. *Medicine* 2023;**102**(20):e33730.

You J, Liu L, Pan X *et al.* Study on the gut microbiota, HPA, and cytokine levels in infantile spasms. *Front Immunol* 2024;**15**:1442677.

Yu F, Ji JL, Wang Y *et al.* Anti-epileptic and gut-protective effects of trioctanoin and the critical role of gut microbiota in a mouse model of epilepsy. *Brain Res Bull* 2025;**227**:111401.

Yuwattana R, Suparan K, Kerdphoo S *et al.* Altered gut microbiome profiles in epileptic children are associated with spectrum of anti-seizure medication responsiveness. *Brain Res* 2025;**1849**:149367.

Żarnowska I, Wróbel-Dudzińska D, Tulidowicz-Bielak M *et al.* Changes in tryptophan and kynurenine pathway metabolites in the blood of children treated with ketogenic diet for refractory epilepsy. *Seizure* 2019;**69**:265–72.

Zeng XD, Hu WG. Spontaneous remission of infantile spasms following rotavirus gastroenteritis. *Neurological Sciences* 2021;**42**(1):253–7.

Zhang Y, Zhou S, Zhou Y *et al.* Altered gut microbiome composition in children with refractory epilepsy after ketogenic diet. *Epilepsy Res* 2018;**145**:163–8.

Zhou C, Gong S, Xiang S *et al.* Changes and significance of gut microbiota in children with focal epilepsy before and after treatment. *Front Cell Infect Microbiol* 2022;**12**:965471.

Zhou K, Jia L, Mao Z *et al.* Integrated macrogenomics and metabolomics explore alterations and correlation between gut microbiota and serum metabolites in adult epileptic patients: a pilot study. *Microorganisms* 2023;**11**(11):2628.

Zierath DK, Davidson S, Manoukian J *et al.* Diet composition and sterilization modifies intestinal microbiome diversity and burden of Theiler's virus infection–induced acute seizures. *Epilepsia* 2024;**65**(6):1777–90.
